# Activation of *HLS1* by Mechanical Stress via Ethylene-Stabilized EIN3 Is Crucial for Seedling Soil Emergence

**DOI:** 10.3389/fpls.2016.01571

**Published:** 2016-10-24

**Authors:** Xing Shen, Yanli Li, Ying Pan, Shangwei Zhong

**Affiliations:** State Key Laboratory of Protein and Plant Gene Research, School of Advanced Agricultural Sciences and School of Life Sciences, Peking UniversityBeijing, China

**Keywords:** seedling emergence, soil, mechanical pressure, apical hook, HLS1, ethylene signaling, EBF1 and EBF2, EIN3/EIL1

## Abstract

The seeds of terrestrial flowering plants often start their life cycle in subterranean darkness. To protect the fragile apical meristematic tissues and cotyledons from mechanical injuries during soil penetration, dicotyledonous seedlings form an elegant apical hook at the top of the hypocotyl. The apical hook has been considered as an adaption structure to the subterranean environment. However, the role of the apical hook in seedling emergence and the molecular mechanism of apical hook formation under real-life conditions remain highly speculative. Here, we find that *HOOKLESS 1* (*HLS1*), a critical gene in apical hook formation in *Arabidopsis thaliana*, is required for seedling emergence from the soil. When grown under soil, *hls1* mutant exhibits severe emergence defects. By contrast, *HLS1* overexpression in the *hls1* background fully restores emergence defects and displays better emergence capacity than that of WT. Our results indicate that *HLS1* transcription is stimulated in response to the mechanical stress of soil cover, which is dependent on the function of the transcription factors ETHYLENE INSENSITIVE 3 (EIN3) and EIN3-LIKE 1 (EIL1). Soil-conferred mechanical stress activates the ethylene signaling pathway to stabilize EIN3 by repressing the activity of the F-box proteins EBF1 and EBF2. These combined results reveal a signaling pathway in which plant seedlings transduce the mechanical pressure of soil cover to correctly modulate apical hook formation during soil emergence.

## Introduction

After seeds are released from the mother plant, they often become covered with leaves and soil, which protects them from adverse environmental conditions including cold temperature and seed predators. When the environment becomes favorable for growth, seeds sense the changes and begin the process of germination (Penfield et al., 2005; Shi et al., 2013, 2015). Germinating seedlings in subterranean darkness undergo heterotrophic growth supported by seed reserves, and direct the apical tip toward the surface. Soil emergence is a particularly vulnerable phase in the life cycle of terrestrial flowering plants, and is tightly controlled by both environmental signals and endogenous hormones (Zhong et al., 2014; de Wit et al., 2016). Buried seedlings have to regulate and adapt their morphology to the surrounding ambient soil environment, which involves darkness and mechanical pressure (Von Arnim and Deng, 1996; Shi et al., 2016). Seedlings grown in the dark display a characteristic phenotype named skotomorphogenesis, which is maintained by central repressors of light signaling, such as CONSTITUTIVELY PHOTOMORPHOGENIC 1 (COP1) (Deng et al., 1991, 1992; Osterlund et al., 1999, 2000). Recent studies show that COP1 is a key component that senses changes in light fluence to correctly modulate the levels of EIN3 proteins during the process of emergence (Shi et al., 2016). Early studies showed that ethylene production is one of the physiological responses to mechanical impedance in pea epicotyls or bean roots (Goeschl et al., 1966; Kays et al., 1974). Subsequent work reported that mutations in key components of the ethylene signaling pathway block seedling soil emergence, which indicates a critical role for ethylene in soil emergence (Harpham et al., 1991).

The plant hormone ethylene is a simple hydrocarbon gas that can be produced in most plant cell types. Although it is chemically simple, ethylene plays vital roles in physiological processes throughout the plant life cycle, including seed germination, seedling emergence, cotyledon greening, fruit ripening, and leaf senescence (Bleecker and Kende, 2000). The most dramatic effect imposed by ethylene on etiolated *Arabidopsis* seedlings is the “triple response,” which is characterized by inhibited root elongation, shortened but thickened hypocotyl, and exaggerated apical hook (Guzman and Ecker, 1990). *Arabidopsis* has been used as a model plant to genetically screen for mutants that fail to display or constitutively display the seedling triple-response phenotype, and most of the key components in ethylene signaling have been identified (Alonso and Stepanova, 2004; Guo and Ecker, 2004). Ethylene is perceived by five endoplasmic reticulum (ER)-localized receptors. In the absence of ethylene, the receptors associate with the negative regulator CONSTITUTIVE TRIPLE RESPONSE1 (CTR1) (Chang et al., 1993; Kieber et al., 1993; Clark et al., 1998), which directly phosphorylates EIN2 and prevents the nuclear translocation of EIN2 C-terminal fragments (Alonso et al., 1999; Ju et al., 2012). The action of CTR1 is repressed in response to ethylene perception. EIN2 C-terminal fragments are cleaved and translocated into the nucleus (Ju et al., 2012; Qiao et al., 2012; Wen et al., 2012). Downstream of EIN2, EIN3 and EIN3-LIKE 1 (EIN3/EIL1) are master transcription factors that conduct essentially all ethylene responses (Chao et al., 1997; Solano et al., 1998; Chang et al., 2013). EIN3 is degraded through the 26S proteasome pathway mediated by two F-box proteins, EBF1 and EBF2 (Guo and Ecker, 2003; Potuschak et al., 2003; Gagne et al., 2004; An et al., 2010). The cleaved EIN2 C-terminus stabilizes EIN3 through at least two methods, by reducing EBF1/EBF2 protein stability and by repressing the translation of *EBF1*/*EBF2* transcripts (Ju et al., 2012; Qiao et al., 2012; Wen et al., 2012; Ji and Guo, 2013; Li et al., 2015; Merchante et al., 2015). Our previous studies showed that EIN3/EIL1 are the key transcription factors involved in mediating seedling soil emergence (Zhong et al., 2014). Ethylene is quantitatively produced in *Arabidopsis* in response to the conditions of soil cover, and EIN3 protein accumulates to high levels in buried seedlings to promote seedling emergence (Zhong et al., 2014). However, it was not clear whether mechanical pressure directly induces EIN3 accumulation via the ethylene signaling pathway.

The “triple response” is proposed to be a morphogenesis strategy adopted by buried seedlings to protect them from mechanical damage when they force their way toward the surface. For example, the apical hook of dark-grown seedlings is believed to protect the shoot apical meristem and cotyledons during penetration through the soil (Harpham et al., 1991; Ecker, 1995; Chen et al., 2005). The apical hook is formed as the result of different cell elongation rates between inner and outer sites, which is driven by an asymmetrical auxin gradient in the hook (Li et al., 2004; Abbas et al., 2013; Mazzella et al., 2014). In addition to cell elongation, cell division also contributes to apical hook development (Raz and Ecker, 1999; Raz and Koornneef, 2001; Vandenbussche et al., 2010; Zadnikova et al., 2010). Forward genetic screening was used to identify a mutant that fails to form the apical hook in dark-grown seedlings, *hookless 1* (*hls1*). HLS1 is essential in regulating apical hook formation (Guzman and Ecker, 1990; Lehman et al., 1996). The auxin response regulators ARF1 and ARF2 act downstream of HLS1 to repress auxin action, whereas environmental light and multiple hormones act upstream of HLS1 to coordinately regulate its activity (Li et al., 2004, 2014; Gallego-Bartolome et al., 2011). Ethylene and gibberellins (GAs) promote *HLS1* action (Lehman et al., 1996; An et al., 2012), whereas light and jasmonate (JA) are negative regulators of HLS1 function (Li et al., 2004; Song et al., 2014; Zhang et al., 2014; Zhu, 2014). Ethylene regulation is mediated through the EIN3/EIL1 transcription factors to directly activate *HLS1* gene expression (An et al., 2012), while GA causes degradation of the repressor DELLA proteins to relieve their repression on EIN3/EIL1 functions (McGinnis et al., 2003; Ariizumi et al., 2008; Wang et al., 2009; An et al., 2012). Exposure to light reduces HLS1 protein levels (Li et al., 2004). Recently, the plant hormone JA was found to reduce *HLS1* gene expression through JA-activated MYC2 repression of EIN3 action (Song et al., 2014; Zhang et al., 2014). These studies suggest that HLS1 represents a central integrator of environmental factors and endogenous hormone signals in controlling apical hook formation.

Although apical hook formation has been considered as an adaptive strategy utilized by etiolated seedlings to penetrate soil, almost all previous studies on apical hook formation and function were performed under soil-free conditions. In this study, we demonstrate that *HLS1* mutation results in the failure of seedling soil emergence. Our results provide clear evidence in support of the hypothesis that soil cover activates *HLS1* gene expression through mechanical pressure-stabilized EIN3 proteins, and is dependent on the ethylene signaling pathway.

## Results

### *HLS1* Mutation Causes Severe Defects in Seedling Soil Emergence

**FIGURE 1:**
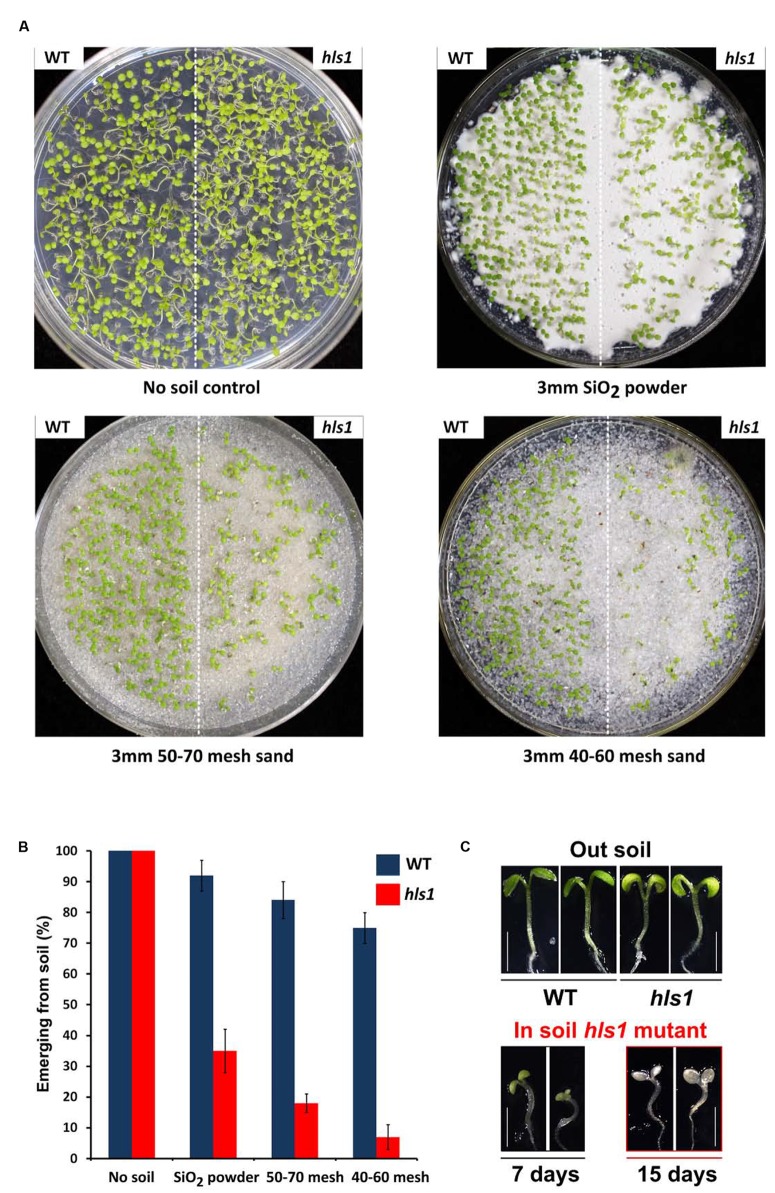
**HLS1 is crucial for plant seedling survival during soil emergence.** **(A,B)** Seedling soil emergence phenotypes **(A)** and quantitative analysis **(B)** of Col-0 (WT) and *hls1* mutant. Seeds were covered with a 3 mm layer of SiO_2_ powder, 50-70 mesh sand particles, or 40-60 mesh sand particles, and then grew under continuous white light for 7 days. Error bars represent SE of three biological replicates (mean ± SE, *n* = 3). **(C)** Phenotypes of WT, *hls1* mutant after emerging from soil (Out soil), and *hls1* mutant remaining in the soil (In soil *hls1* mutant). Seedlings grew for 7 or 15 days on the plates with a 3 mm layer of 50-70 mesh sand particles under continuous white light.

To determine whether the apical hook functionally promotes seedling soil emergence, we examined the emergence phenotypes of *A. thaliana* Col-0 (WT) and *hls1* mutant seedlings when covered by a layer of soil. In the assay, we first sowed seeds on half-strength MS agar medium, and then covered them evenly with various types of soil, including SiO_2_, 50-70 mesh sand, and 40-60 mesh sand. Seedlings grew in continuous white light for 7 days, and the emergence phenotypes and frequencies were recorded. We found that WT and *hls1* mutant grew normally in the absence of soil cover (**Figure 1A**). When covered by a 3 mm layer of soil, more than 70% of WT seedlings emerged from the soil. Increasing the particle size of the soil cover gradually reduced WT seedling emergence frequencies (**Figures 1A,B**). By contrast, the *hls1* mutant exhibited severe emergence defects under soil (**Figure 1C**): the emergence frequency dramatically decreased to less than 10% with increasing soil particle size (**Figures 1A,B**). The *hls1* mutant seedlings remaining under soil cover displayed bleaching phenotypes and eventually died (**Figure 1C**). These results indicate that HLS1 is required for seedling survival during soil emergence.

### HLS1 Overexpression Enhances Seedling Soil Emergence

**FIGURE 2:**
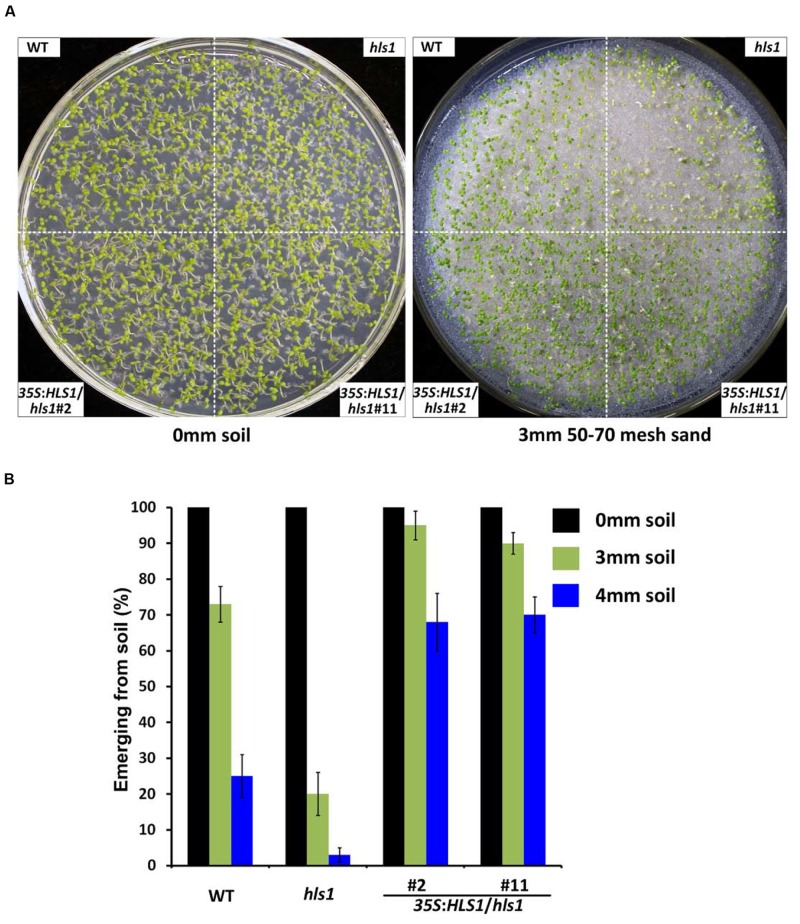
***HLS1* overexpression in the *hls1* mutant fully restores and enhances seedling emergence from soil.** **(A,B)** Seedling soil emergence phenotypes **(A)** and quantitative analysis **(B)** of WT, *hls1*, and two independent lines overexpressing *HLS1* in the *hls1* mutant background (*35S*:*HLS1*/*hls1* #2 and #11). Seedlings grew for 7 days on the plates without soil cover (0 mm soil), with a 3 mm or 4 mm layer of 50-70 mesh sand particles under continuous white light. Mean ± SE, *n* = 3.

In the complementation experiment, we used two independent transgenic lines overexpressing full-length *HLS1* to introduce *HLS1* into the *hsl1* mutant background. The transgenic *35S*:*HLS1*/*hls1* seedlings have been shown to rescue the hookless phenotype of the *hls1* mutant, and exhibit enhanced hook formation in darkness compared with that of WT (An et al., 2012). In our soil assay, WT, *hls1*, and *35S:HLS1*/*hls1* seedlings grew well under continuous white light in the absence of soil cover (**Figure 2A**). When covered by a 3 mm layer of 50-70 mesh sand, most *hls1* mutant seedlings did not emerge from the soil, whereas HLS1 overexpression fully rescued the emergence defects of the *hls1* mutant to display even better soil emergence than WT (**Figures 2A,B**). When the seedlings were covered by a 4 mm soil layer, the emergence frequency of WT decreased to 30% and less than 10% of *hls1* mutant could emerge (**Figure 2B**). However, the *35S*:*HLS1*/*hls1* still maintained high soil emergence frequencies of approximately 70% (**Figure 2B**). These combined results demonstrate that HLS1 is essential for mediating seedling emergence from the soil.

### Soil Covering-Conferred Mechanical Stress Stimulates *HLS1* Gene Expression

**FIGURE 3:**
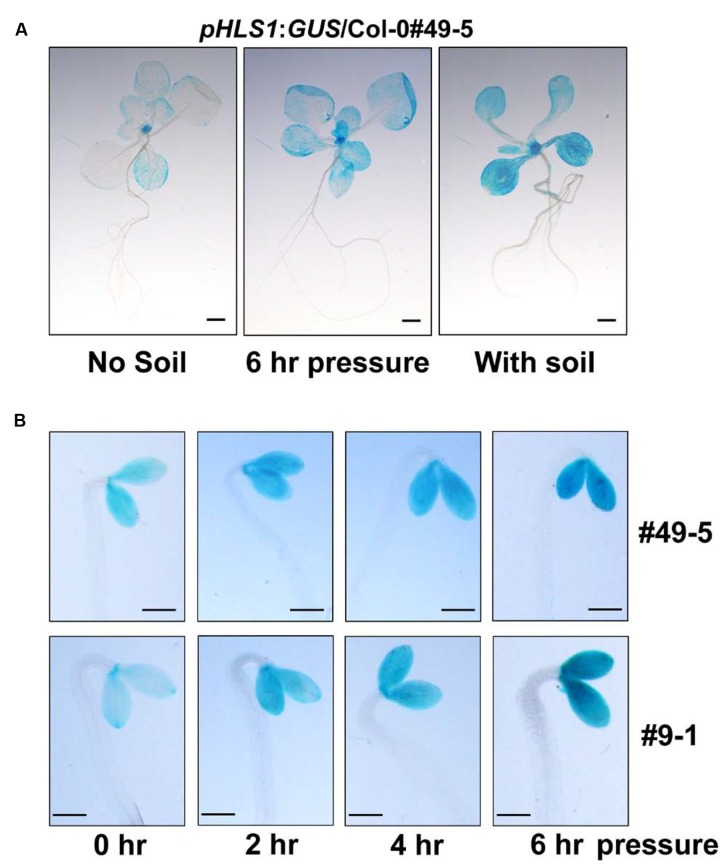
***HLS1* gene transcription is activated by soil cover or mechanical pressure.** **(A)** GUS staining of 12-day-old light-grown transgenic seedlings expressing p*HLS1*:*GUS* in the Col-0 (WT) background. Seedlings grew on the plates without soil cover (No soil), were subjected to mechanical stress by the pressure of a glass plate (∼150 Pa, 6 h pressure), or emerged from 3 mm of 40-60 mesh sand particles (With soil). Scale bar = 1 mm. **(B)** GUS staining of 4-day-old dark-grown seedlings expressing p*HLS1*:*GUS* in the Col-0 (WT) background. Two independent p*HLS1*:*GUS*/Col-0 transgenic lines (#49-5 and #9-1) were used in the study. Etiolated seedlings were subjected to mechanical stress by the pressure of a glass plate (∼150 Pa) in the dark for the indicated time periods and then stained for GUS activity analysis. Scale bar = 1 mm.

Next, we investigate how HLS1 is regulated during seedling soil emergence. We used *pHLS1*:*GUS*/Col-0 transgenic lines, in which the *HLS1* promoter drives β-glucuronidase reporter gene expression, to visualize *HLS1* gene expression by detecting GUS activity (Zhang et al., 2014). We grew *pHLS1*:*GUS*/Col-0 transgenic seedlings side-by-side under continuous white light with and without soil cover. The GUS staining results showed that *HLS1* gene expression levels were obviously elevated in the presence of soil cover (**Figure 3A**). As soil cover can cause mutiple effects to the seedlings at the same time, we examined mechanical stress-regulated *HLS1* gene expression by placing a transparent glass plate on the seedlings to exert pressure, and found that *HLS1* gene expression was upregulated in apical meristem and cotyledons, similar to that observed with soil cover (**Figure 3A**). To confirm the regulation of *HLS1* gene transcription by mechanical stress, we performed a time-course GUS staining experiment in the dark. We used a glass plate to press against 4-day-old etiolated seedlings and detected GUS activity at different time points. We found that *HLS1* was expressed in both apical meristem and cotyledons of the etiolated seedlings (**Figure 3B**). Mechanical stress induced an increase in *HLS1* gene expression levels within 2 h, which gradually increased and then reached stable levels after 6 h (**Figure 3B**). Two independent lines exhibited similar *HLS1* gene expression patterns in response to mechanical stress (**Figure 3B**). These results suggest that *HLS1* is induced by soil-conferred mechanical stress at the transcriptional regulation level.

### Mechanical Pressure-Induced *HLS1* Gene Expression Is Dependent on EIN3/EIL1

**FIGURE 4:**
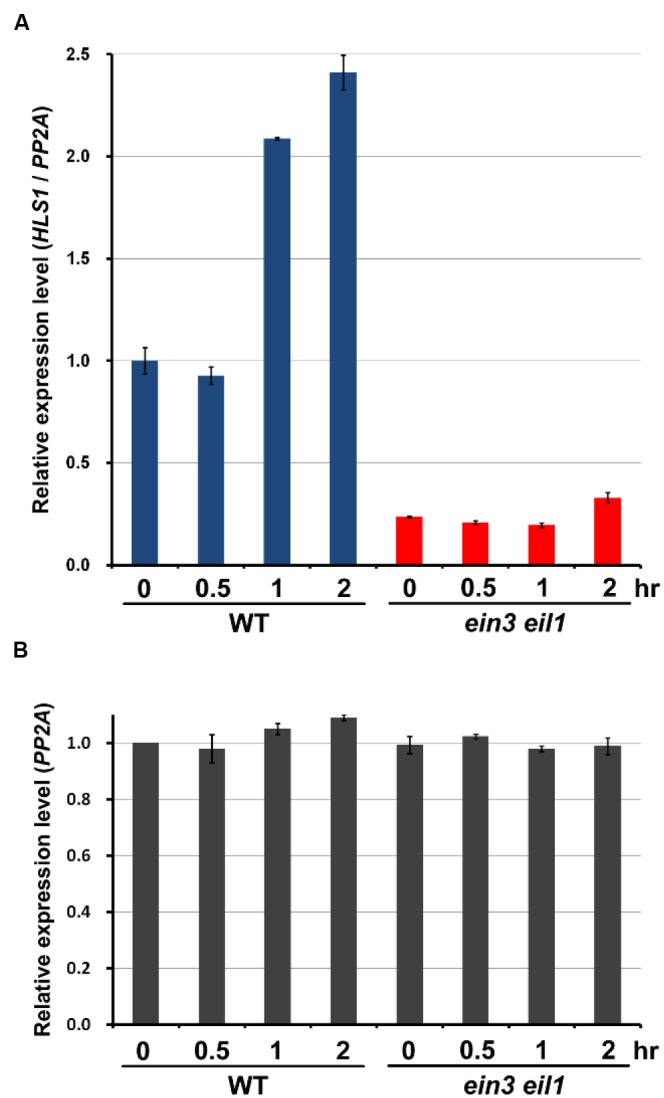
**Mechanical pressure-stimulated *HLS1* gene expression is dependent on EIN3/EIL1 function.** **(A,B)** Real time RT-PCR results show the *HLS1* gene expression levels in 4-day-old dark-grown Col-0 (WT) and *ein3 eil1* mutant seedlings subjected to approximately 150 Pa mechanical stress for the indicated time periods. *PP2A* was analyzed as an internal control (*PP2A*). *HLS1* expression was normalized with respect to that of *PP2A* (*HLS1*/*PP2A*) **(A)**. Relative *PP2A* expression levels in different samples, in which the basal *PP2A* expression level in WT without mechanical stress treatment was set as 1 **(B)**. Mean ± SD, *n* = 3.

Previous studies showed that *HLS1* gene expression is activated by the transcription factors EIN3/EIL1 (An et al., 2012). We hypothesized that mechanical stress-induced *HLS1* gene expression also might be mediated through EIN3/EIL1. We conducted quantitative RT-PCR (qRT-PCR) to analyze *HLS1* transcript levels in response to mechanical stress in WT and the *ein3eil1* mutant. Consistent with the GUS staining results, qRT-PCR results for WT seedlings showed that *HLS1* gene expression was significantly induced after 1 h of mechanical stress conferred by the pressure of the glass plate (**Figure 4**). However, in the *ein3eil1* mutant, *HLS1* gene expression levels were reduced to less than half of that in WT without mechanical stress (**Figure 4**), which is consistent with EIN3 activation of *HLS1* gene expression. Moreover, *HLS1* gene expression levels in the *ein3eil1* mutant did not respond to mechanical stress, and were maintained at low levels even within 2 h of mechanical stress (**Figure 4**). These results indicate that EIN3/EIL1 are required for mechanical stress-induced *HLS1* gene expression.

### Mechanical Stress Stabilizes EIN3 Protein Levels via the Ethylene Signaling Pathway

**FIGURE 5:**
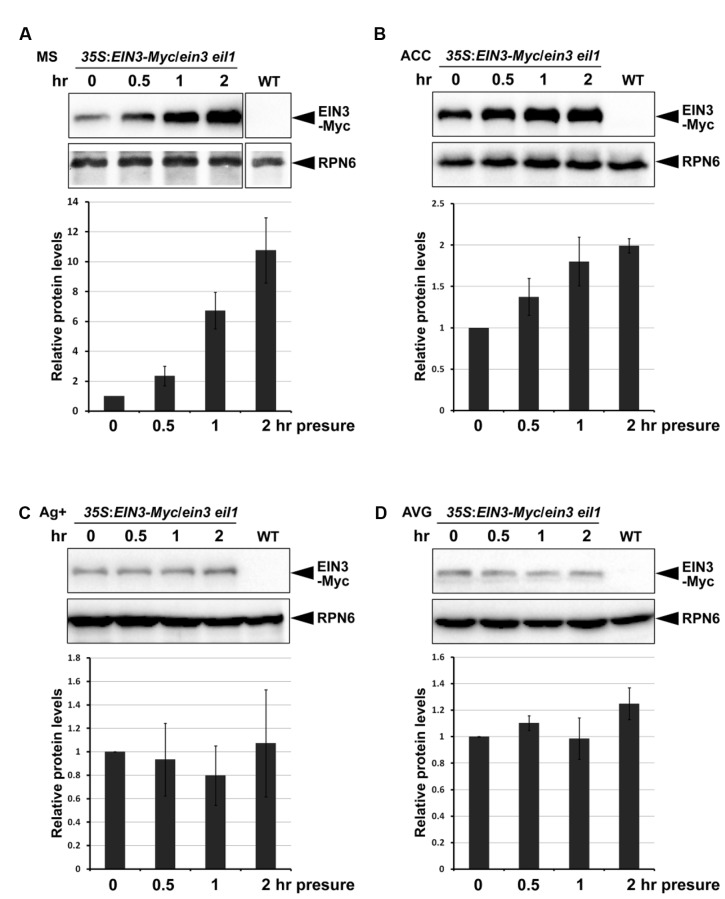
**EIN3 protein levels are stabilized by mechanical stress through the ethylene signaling pathway.** **(A-D)** Western blot results show the EIN3 protein levels in transgenic plants constitutively expressing EIN3-Myc fusion proteins in the *ein3 eil1* background. Seedlings grew for 4 days on half-strength MS medium **(A)**, half-strength MS medium supplemented with 10 μM ethylene precursor ACC **(B)**, half-strength MS medium supplemented with 100 μM ethylene perception inhibitor Ag^+^ **(C)**, or half-strength MS medium supplemented with 25 μM ethylene biosynthesis inhibitor AVG **(D)** in the dark. Seedlings were then subjected to approximately 150 Pa mechanical stress for the indicated time periods before performing protein analysis. Bottom panels show the results of quantification analysis of EIN3-Myc protein. EIN3-Myc protein levels were normalized with respect to the control protein RPN6, and the initial EIN3-Myc protein levels without mechanical stress in each experiment were set as 1. Mean ± SE, *n* = 3.

Previous studies showed that EIN3 is a key transcription factor in mediating seedling soil emegence (Zhong et al., 2014). EIN3 protein levels are gradually reduced when seedlings aproach the surface. The COP1-EBF1/EBF2-EIN3 tandem E3 ligase mechanism senses light changes during soil emergence. However, the promotion of EIN3 protein accumulation by soil-conferred mechanical stress is independent of COP1 (Shi et al., 2016). Given the evidence that mechanical stress induces ethylene production (Goeschl et al., 1966), and ethylene stabilizes EIN3 proteins (Guo and Ecker, 2003), it is assumed that soil-conferred mechanical stress induces EIN3 protein accumulation through ethylene signaling. To verify this hypothesis, we examined the effects of the ethylene pathway on EIN3 protein levels in response to mechanical stress. Etiolated seedlings grew for 4 days on half-strength MS medium (control), or half-strength MS medium containing either the ethylene precursor ACC, the ethylene perception inhibitor Ag^+^, or the ethylene biosynthesis inhibitor AVG. Then, the etiolated seedlings were subjected to mechanical stress by pressing with a glass plate. Our results showed that EIN3 protein levels were markedly elevated within 0.5 h of mechanical stress, and displayed an approximately 10-fold increase after 2 h of mechanical stress on half-strength MS medium (**Figure 5A**). ACC treatment resulted in constitutively higher EIN3 protein levels without mechanical stress, and a much slower rate of EIN3 protein increase in response to mechanical stress (**Figure 5B**). By contrast, Ag^+^ suppressed ethylene perception, reduced EIN3 protein levels compared with those on half-strength MS medium, and largely repressed seedling responses to mechanical stress (**Figure 5C**). AVG treatment blocked endogenous ethylene biosynthesis, and thereby blocked EIN3 protein accumulation in response to mechanical stress (**Figure 5D**). These combined results indicate that the ethylene signaling pathway is requried for mechanical stress-stabilized EIN3 accumulation.

### EBF1 and EBF2 Are Required for Increased EIN3 Protein Levels in Response to Mechanical Stress

**FIGURE 6:**
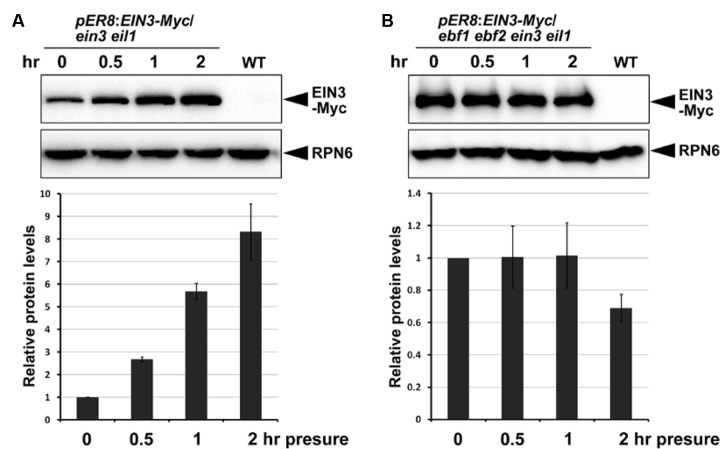
**F-box proteins EBF1 and EBF2 are required for elevation of EIN3 protein levels in response to mechanical stress.** **(A,B)** Western blot results show the EIN3 protein levels in transgenic plants expressing inducible EIN3-Myc fusion proteins in the *ein3 eil1* **(A)** and *ebf1 ebf2 ein3 eil1* **(B)** backgrounds. Seedlings grew for 4 days on half-strength MS medium supplemented with 10 μM β-estradiol in the dark, and were then subjected to approximately 150 Pa mechanical stresses for the indicated time periods before performing protein analysis. Bottom panels show the results of quantification analysis of EIN3-Myc protein. EIN3-Myc protein levels were normalized with respect to the control protein RPN6, and the initial EIN3-Myc protein levels without mechanical stress in each experiment were set as 1. Mean ± SE, *n* = 3.

Previous studies reported that EIN3 degradation was dependent on the F-box proteins EBF1 and EBF2 through the 26S-proteasome-mediated degradation pathway (Guo and Ecker, 2003; Potuschak et al., 2003; Gagne et al., 2004), and ethylene inhibited the actions of EBF1/EBF2 at multiple levels to stabilize EIN3 (An et al., 2010; Ju et al., 2012; Qiao et al., 2012; Wen et al., 2012; Li et al., 2015; Merchante et al., 2015). Therefore, we evaluated whether EBF1/EBF2 mediate increased EIN3 protein levels in response to mechanical stress. We introduced an inducible EIN3-Myc fused protein in the *ein3 eil1* (with EBF1/EBF2) and *ebf1 ebf2 ein3 eil1* (without EBF1/EBF2) mutant backgrounds. EIN3-Myc proteins were induced by β-estradiol treatment, and the seedlings were then subjected to mechanical stress by a glass plate. Our results indicated that EIN3 protein levels were dramatically increased after mechanical stress when EBF1/EBF2 were present (**Figure 6A**), but remaining unchanged in the absence of EBF1/EBF2 function (**Figure 6B**). In light of these combined results, we propose that soil-conferred mechanical stress induces EIN3 protein accumulation through the ethylene-SCF^EBF1/EBF2^ signaling pathway.

## Discussion

**FIGURE 7:**
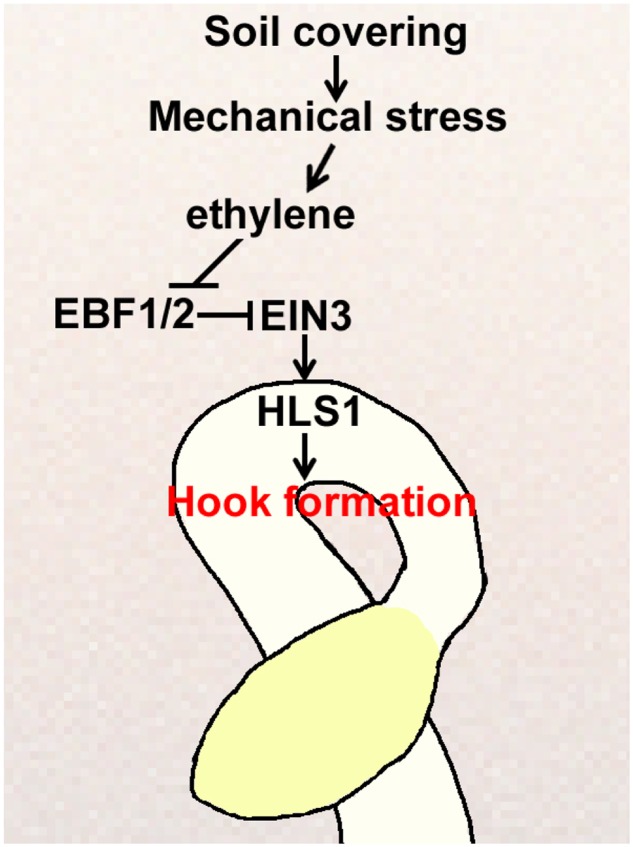
**Proposed model for how soil cover induces apical hook formation in plant seedlings.** Soil cover causes mechanical stress, which stimulates ethylene production, and then activates *HLS1* gene expression through the SCF^EBF1/EBF2^-EIN3 pathway to correctly modulate seedling apical hook formation.

Plants undergo physiological and developmental changes in response to mechanical pressure. The apical hook is a unique functional structure that forms in dark-grown etiolated seedlings and is greatly exaggerated by the plant hormone ethylene, which is strongly induced by mechanical stress (Goeschl et al., 1966; Guzman and Ecker, 1990; Lehman et al., 1996; Zhong et al., 2014). Historically, it has been assumed that the apical hook protects the apical meristematic tissues and cotyledons of germinating seedlings when penetrating the soil (Guzman and Ecker, 1990). However, the mechanism that regulates apical hook formation in real soil conditions is rather fragmented. In this study, we showed that apical hook formation was regulated by soil cover through a linear signaling pathway, and played a pivotal role in mediating seedling emergence from the soil (**Figure 7**). *HLS1* mutation resulted in defective apical hook formation and caused severe defects in seedling soil emergence, which were fully restored by *HLS1* overexpression in the *hls1* mutant. We also showed that soil cover caused mechanical stress, which activated *HLS1* gene expression dependent on the ethylene-SCF^EBF1/EBF2^-EIN3 signaling pathway. When buried seeds germinate in soil, seedlings produce ethylene gas in response to the mechanical compression of soil, and ethylene represses the action of the E3 ligase complexes SCF^EBF1/EBF2^ to stabilize EIN3, which activates *HLS1* gene expression. As a result, apical hook formation is regulated according to the mechanical stress conferred by the specific soil cover conditions.

Seedling emergence is one of the most critical stages in the plant life cycle. Early studies showed that ethylene evolution dramatically increased when pea epicotyls or bean roots were subjected to physical impedance (Goeschl et al., 1966; Kays et al., 1974). Subsequent work used *Arabidopsis* mutants with impaired ethylene responses, and showed that the ethylene signaling pathway was essential for seedling emergence through sand cover (Harpham et al., 1991; Zhong et al., 2014; Shi et al., 2016). Our recent studies show that germinating seedlings produce ethylene in accordance with the specific soil conditions and concurrently activate two tissue-specific genes through EIN3, *ETHYLENE RESPONSE FACTOR 1* (*ERF1*) and *PHYTOCHROME INTERACTING FACTOR 3* (*PIF3*) (Zhong et al., 2012, 2014). Soil-conferred mechanical stress stimulates ERF1 action in the hypocotyl to modulate cell elongation, whereas the action of PIF3 is co-activated in the cotyledon to adjust protochlorophyllide biosynthesis (Zhong et al., 2014). Coupling of these two components synchronizes the rates of hypocotyl growth and etioplast maturation, which enables seedlings to maintain suitable etioplast development to rapidly achieve photoautotrophic capacity without causing photooxidative damage on reaching light at the surface. In this study, we identified a novel signal transduction pathway, in which soil overlay promotes apical hook formation through ethylene-EBF1/EBF2-EIN3-activated *HLS1* gene expression. Therefore, EIN3 acts as an integrating hub that transduces the information of soil cover to simultaneously adjust hypocotyl growth, cotyledon development, and apical hook formation via different downstream pathways.

Seedlings growing under soil have to sense and integrate multiple environmental cues to grow out. Perception and response to mechanical stimuli are vital for seedling survival. Seedlings have to sense the mechanical pressure and relay the information to adjust their morphogenesis (Braam and Davis, 1990; Braam, 2005; Chehab et al., 2009). Recent studies reported that both JA and GA are important in mediating touch-induced changes in adult plants morphology (Chehab et al., 2012; Lange and Lange, 2015), suggesting the important roles of JA and GA in thigmomorphogenesis. Seedling response to gravity is another critical characteristic for seedling growth under soil. Ethylene and brassinosteroids (BR) act antagonistically in controling gravitropism. In root, ethylene reduces gravitropic bending responses, whereas BR enhance these responses. By contrast, in shoot, ethylene promotes gravitropic growth, whereas BR represses gravitropism (Buer et al., 2006; Guo et al., 2008; Li, 2008; Vandenbussche et al., 2011). Further studies reported that both ethylene and BR control shoot gravitropism largely through the regulation of auxin signaling components and auxin distribution (Gupta et al., 2012; Vandenbussche et al., 2013). As shoot gravitropism is essential for seedling growth upward after underground germination, it is therefore likely that BR and auxin also are involved in mediating seedling soil emergence. Besides mechanical stress and gravity, it has been recently demonstrated that hypoxic conditions that seedlings might encounter in the underground environment constitute an important environmental cue promoting hook development (Abbas et al., 2015; Zhu and Benkova, 2016). It will be important to investigate the interplay of environmental signals and various plant hormones in regulating the process of seedling emergence.

In addition to conferring mechanical stress, soil overlay also causes a dark environment. Dark-grown seedlings form an apical hook at the meristem, and unfurl the apical hook and cotyledons on light exposure. Previous studies showed that COP1 mutation abolished hook formation in etiolated seedlings, suggesting that COP1 was indispensable for maintaining the apical hook in the dark (Deng et al., 1991). A recent study found that COP1 is the E3 ligase of EBF1/EBF2, which stabilizes EIN3 accumulation by mediating EBF1/EBF2 protein ubiquitination and degradation (Shi et al., 2016). Based on these combined results, we propose that light triggers hook opening by repressing COP1 activity and reducing EIN3-activated *HLS1* transcription. However, EIN3 overexpression in *cop1* mutant only partially restores apical hook phenotypes, which suggests that there are other regulators mediating hook formation downstream of COP1 (Shi et al., 2016). Phytochrome interacting factors (PIFs) are a group of bHLH transcription factors that play central roles in mediating light responses (Leivar and Quail, 2011; Zhang et al., 2013; Pfeiffer et al., 2014). Given that PIF mutations result in apical hook unfolding, and COP1 stabilizes PIF proteins in the dark, it is likely that PIFs act downstream of COP1 to promote apical hook formation (Bauer et al., 2004; Leivar et al., 2008, 2009; Zhu et al., 2015; Pacin et al., 2016). Therefore, EIN3 and PIFs represent two key transcription factors modulating apical hook formation in response to mechanical stress and light signals. Future efforts to identify PIF regulation of HLS1 will help to reveal the underlying mechanism of apical hook development during the process of seedling soil emergence.

## Materials and Methods

### Plant Materials and Growth Conditions

Wild-type (WT) *Arabidopsis thaliana* Columbia-0 (Col-0) ecotype seedlings were used in this study. The *hls1-1* (Lehman et al., 1996), *35S*:*HLS1*/*hls1-1* (An et al., 2012), *ProHLS1-GUS*/Col-0 (Zhang et al., 2014), *ein3 eil1* (Zhong et al., 2009), and *35S*:*EIN3-Myc*/*ein3 eil1* (Shi et al., 2016) plants were reported previously. Seeds were surface sterilized with 75% ethanol containing 0.1% Triton X-100 for 15 min, and then washed three times with sterile water. Sterile seeds were plated on half-strength MS medium (2.2 g/l MS salts, 0.5% sucrose, and 8 g/l agar, pH = 5.7). Plates were stratified in darkness at 4°C for 3 days, and then transferred to grow under white light at 22°C for the indicated periods of time.

### Soil Emergence Assay and Mechanical Stress Treatment

For soil emergence assays, we grew all plants and harvested seeds side-by-side. Surface-sterilized seeds were plated on half-strength MS medium. Silicon dioxide (SiO_2_) powder and sand (Sigma) were used as soil cover after they were sterilized by autoclaving (Zhong et al., 2014; Shi et al., 2016). The autoclaved powder or sand was measured using a 50-mL Falcon tube, and then evenly spread onto the MS agar medium containing plated seeds. The plates were then placed in the dark at 4°C for 2 days for stratification before transferring to white-light illumination for 6 h to induce germination. The plates were then incubated under white light or in darkness for the indicated number of days. At least 150 seeds were used for each experimental treatment, and three biological replicates were used for statistical analysis.

The 4-day-old etiolated seedlings or 7-day-old green seedlings were subjected to mechanical stress treatment for the indicated time. One circular transparent glass plate was plated onto the top layer of the Petri dishes to induce mechanical stress of approximately 150 Pa onto seedlings.

### GUS Staining Assay

The GUS staining assay was performed as described previously (Zhong et al., 2010) with minor modification. The 4-day-old etiolated seedlings or 12-day old light-grown seedlings were immersed in the GUS staining solution (1 × PBS, 1 mM K_3_Fe(CN)_6_, 0.5 mM K_4_Fe(CN)_6_, 1 mM EDTA, 1% Triton X-100, and 1 mg/ml X-gluc), and incubated at 37°C in the dark for 20 min to several hours depending on the experimental requirements. The seedlings were then destained in 90 and 70% ethanol. The stained samples were observed using a Leica Microsystems DFC295 microscope.

### RNA Extraction and qRT-PCR

The 3-day-olds dark-grown etiolated seedlings were subjected to mechanical stress conferred by a glass plate (pressure ∼150 Pa) for the indicated period of time. Seedlings were then harvested and ground to powder in liquid nitrogen. Total RNA was extracted using the Spectrum Plant Total RNA Kit (Sigma). Spectrophotometric and gel electrophoretic analyses were performed to determine RNA quality. To synthesize cDNA, 1 μg of RNA was used for reverse transcription using ReverTra Ace qPCR RT Kit (TOYOBO). Real-time PCR was performed using SYBR Green Mix (Takara) and the ABI fast 7500 Real-Time system. The gene expression results were normalized with respect to *PP2A* expression levels. All quantitative PCR experiments were independently performed in triplicate, and representative results are shown. The following primers were used:

RTM-PP2A-F, 5′-GTGACTTGGTTGAGCATTTCACTCC-3′;
RTM-PP2A-R, 5′-GAGCTGATTCAATTGTAGCAGCAAACT-3′;
RTM-HLS1-F, 5′-CACGGTTATCAAGTTAGAGC-3′;
RTM-HLS1-R, 5′-GAAAGTCCCAAGCGAGA-3′.

### Protein Western Blot

Surface-sterilized 35S:EIN3-Myc/*ein3eil1* seeds were plated on half-strength MS medium, half-strength MS medium containing 10 μM ACC, or half-strength MS medium containing 100 μM AgNO_3_. Seeds of *pER8*:EIN3-Myc/*ebf1ebf2ein3eil1* were plated on half-strength MS medium containing 10 μM β-estradiol to induce EIN3-Myc expression. After treatment at 4°C in the dark for 2 days, the plates were irradiated with white light for 6 h to induce germination. Then, the plates were incubated in dark conditions for 4 days, and the etiolated seedlings were subjected to mechanical pressure conferred by an overlying glass plate with pressure of approximately 150 Pa for the indicated time in the dark. Subsequently, the seedlings were harvested in liquid nitrogen and ground to powder. The anti-Myc (Sigma, 1:2000 dilution) and anti-RPN6 (Millipore, 1:5000 dilution) antibodies were used for immunoblotting analysis.

## Author Contributions

SZ designed the research; XS, YL, and YP performed the experiments; SZ, YL, XS, and YP analyzed the data, prepared the figures, and SZ wrote the manuscript.

## Conflict of Interest Statement

The authors declare that the research was conducted in the absence of any commercial or financial relationships that could be construed as a potential conflict of interest.
